# Correlation analisys of nursing outcomes classification (NOC) to diagnosis related groups (DRG) in critically ill patients (CIP)

**DOI:** 10.1186/2197-425X-3-S1-A959

**Published:** 2015-10-01

**Authors:** J Ruiz Moreno, MJ Esteve Paños, E González Marín, M Moral Guiteras, N Suárez Álvarez, M Juliá Amill, R Corcuera Romero de la Devesa, F Baigorri González, A Artigas Raventós

**Affiliations:** QuirónSalud Hospital Universitario Sagrat Cor, Critical Care Department, Barcelona, Spain; Hospital de Sabadell & QuirónSalud Hospital Universitario Sagrat Cor, Critical Care Department, Sabadell, Spain

## Introduction

Despite attending the same patient, in the same place (ICU) and at the same time, we establish the hypothesis that the NOC are not related to medical diagnostics in reference to critically ill patients (CIPs); medical diagnostics understood as DRG.

## Objectives

• In regard to CIPs, to analyze and evaluate which DRG involve a greater nº of NOCs.

• To analyze the correlation between the relative weight (RW) of the DRGs and the number of NOCs.

## Methods

• Type of Study: prospective, analytical, longitudinal, and observational.

• First period: December 10-2012 - May 22-2013.

• Second period: August 19-2013 - June 30-2014.

• Setting : Medical / surgical ICU belonging to a 275 acute care teaching hospital.

• Population and sample: All the CIPs (878) admitted consecutively to the ICU. Sample: 760 CIPs.

• Exclusión criteria: CIPs < 16 years, major burn patients, incomplete clinical documentation, and voluntary discharge.

• DRG AP-DRG 25.0 version (684 DRG are grouped into 25 Major Diagnostic Categories and 1 extra Category)

• NOC 5th ed. (490 results are grouped into 32 clases and 7 domains. Specific CCN outcomes (SCCNO) are identified.

• Specific critical care nursing outcomes (SCCNO) are identified.

• Coeficiente correlación lineal de Pearson (r).

• Data collection: 6 registered critical care nurses trained in case-mix.

• Statistical analysis: ANOVA, 'F' of Snedecor. Scheffe's test. Pearson's correlation coefficient (Pearson's *r*)

## Results

• 878 CIPs, that are assigned to 106 diferent DRGs.

• SCCN**O** identified: 212 NOCs

• 46 DRGs include 5 or > CIPs, encompassing 760 CIPs (118 cases are assigned to DRG including 4 or < CIPs)

• Number of NOC per DRG: 341.86 (DE = 30.1)

• NOC average per DRG: 341.86 (confidence interval 0.05, SD = 30.1)

• ANOVA significativo (F Snedecor = 1.709, p = 0.003).

• Scheffe's test is used to detect the DRGs incorporating a greater or lesser number of NOC. Scheffe's test is applies in 89 combinations or pairs of 2 DRGs: 261 pairs (27,8 %) are significant.

• 6 DRGs show significant differences with more than 20 DRGs: 290, 353, 540, 585, 837 y 882:

- < number of NOCs: DRG 290 (326 NOC) y DRG 837 (322 NOC)

- > number of NOCs: DRG 353 (357 NOC), DRG 540 (354 NOC), DRG 585 (353 NOC), y DRG 882 (357 NOC).

• 1 DRG (568) shows no significant difference in relation to the rest: 340 NOCs.

• Pearson's correlation coefficient between RW and nº of NOC: r = 0.21. So, a greater RW does not involve a greater number of NOCs. Some DRGs comprises a different number of NOCs than others (not sure if > or < ).

## Conclusions

• The distribution of NOCs by DRG is not uniform: high RW do not involve a greater number of NOCs.

• The NOCs are not correlated with the DRGs. In other words, although the CIP is the same, is attended in the same area (ICU), nursing outcomes (NOC) are not associated with medical diagnostics incorpored into the DRGs.

